# Differential misclassification of confounders in comparative evaluation of hospital care quality: caesarean sections in Italy

**DOI:** 10.1186/1471-2458-14-1049

**Published:** 2014-10-08

**Authors:** Mirko Di Martino, Danilo Fusco, Paola Colais, Luigi Pinnarelli, Marina Davoli, Carlo Alberto Perucci

**Affiliations:** Department of Epidemiology, Regional Health Service, Lazio Region, Via di Santa Costanza, 53-00198 Rome, Italy; National Agency of Regional Health Services, Rome, Italy

**Keywords:** Differential misclassification of confounders, Comparative effectiveness research, Caesarean section, Hospital care quality

## Abstract

**Background:**

Despite extensive studies on exposure and disease misclassification, few studies have investigated misclassification of confounders. This study aimed to identify differentially misclassified confounders in a comparative evaluation of hospital care quality and to quantify their impact on hospital-specific risk-adjusted estimates, focusing on the appropriateness of caesarean sections (CS).

**Methods:**

We gathered data from the Hospital Information System in Italy for women admitted in 2005–2010. We estimated adjusted proportions of CS with logistic regression models. Among several confounders, we focused on high fetal head at term (HFH), which is seldom objectively documentable in medical records.

**Results:**

A total of 540 maternity units were compared. The median HFH prevalence was 0.9%, ranging from 0 to 70%. In some units, HFH was coded so frequently that it was unlikely to reflect a natural heterogeneity. This “over-coding” was conditional on the outcome because it occurred more frequently for women that underwent CS. This suggested an opportunistic coding to justify the choice of a CS. HFH misclassification was not randomly distributed over Italy; it had an excess in the Campania region where, in some units, the proportion of HFHs gradually increased from 2005 to 2010 (e.g., from 0 to 26%), but the national average remained constant (2.5%). The inclusion of the misclassified diagnosis in the models favored those hospitals that codified in a less-than-fair manner.

**Conclusions:**

Our findings emphasized the importance of rigorously inspecting for differential misclassification of confounders. Their validity may be subject to substantial heterogeneity over hospitals, over time and geographical areas.

**Electronic supplementary material:**

The online version of this article (doi:10.1186/1471-2458-14-1049) contains supplementary material, which is available to authorized users.

## Background

Despite extensive studies on exposure and outcome misclassification in epidemiology, few studies have focused on the misclassification of confounders; nevertheless, the resulting bias may be quite relevant and misleading. In general terms, a misclassified confounding variable hinders the ability to control confounding. However, nondifferential and differential misclassification lead to substantially different consequences. A nondifferential misclassification will reduce the degree to which the confounder can be controlled. This bias can be viewed as a residual confounding problem. The result would be expected to lie between the unadjusted association and the “true”, correctly-adjusted association, which would have been obtained if the confounder had not been misclassified [1, 2]. In contrast, a differential misclassification cannot be considered a residual confounding problem, because additional distortion may lead to unpredictable consequences with respect to the magnitude and direction of bias in the “adjusted” estimates [3].

In this study, we aimed to describe and investigate the impact of differential misclassification of confounders within the framework of a comparative analysis of hospital care, focusing on caesarean sections (CS) in Italy. In 2009, the Italian CS rate (38.4%) was among the highest in the world [4]. The increase in national CS rates tended to slow down over the last few years, both for women that had not had a previous caesarean delivery (*primary*) and for women that had undergone a previous caesarean delivery (*repeated*). Primary caesarean deliveries, which comprise 2/3 of the overall CS rate, are an important target for reduction, because they lead to increased risk for a repeat caesarean delivery [5–7]. Therefore, some authors have suggested that we should focus on primary CSs for inter-hospital comparisons and quality improvements [8].

International concern over increases in CS deliveries have prompted the World Health Organization to suggest that cesarean delivery rates should not exceed 10% - 15% of the total number of births that occur in industrialized countries. Rates above this threshold could be considered inappropriate, and maternal and neonatal benefits may no longer outweigh the costs and risks associated with this procedure [9].

Therefore, CS rates are one of the most frequently used indicators of health care quality. Hospitals, and more generally, health-care systems, may be compared on the basis of this indicator, with the implicit assumption that lower CS rates reflect more appropriate health care practice. However, many studies have emphasized that comparisons may be methodologically biased and misleading to the public when they fail to account for factors related to the increased likelihood of CSs, such as maternal age, fetal distress, placental abnormalities, comorbidities, and other risk factors [10–13]. Malposition and malpresentation of the fetus are among the most important reasons for performing a CS [14].

On one hand, risk adjustment methodologies are essential for obtaining valid estimates; however, on the other hand, improper definition of confounders may introduce further unexpected biases and provide a distorted picture of reality. This methodological fallacy may be particularly relevant when clinical factors, that are an indication for caesarean delivery, are subject to differential misclassification. In some cases, a differential misclassification may reflect opportunistic diagnosis coding, in an attempt to justify the choice of a CS in the absence of actual risk factors. This problem is particularly marked in diagnoses that are seldom objectively documentable in medical records. Some malpositions and malpresentations of the fetus are commonly affected by this problem, particularly the diagnosis of high fetal head at term (HFH).

The objectives of this study were to check for possible differential misclassification of confounders in a comparative evaluation of hospital care and to quantify the impact of misclassification on hospital-specific risk-adjusted estimates, with a focus on the appropriateness of CSs.

## Methods

### Data sources and study population

Data were collected within the framework of the National Outcome Program, currently active in the Italian Health System. This program, introduced in 2010, performs comparative analyses of hospital care, and more than 100 outcome indicators of inpatient care are evaluated [15]. The results provided by the National Outcome Program are updated every year and are publicly available, including the data analyzed in this study [16]. We collected data from all infant deliveries in Italy from January 1, 2005 through December 31, 2010 from the Hospital Information System. The data included demographics (sex, date, and place of birth, place of residence), admission and discharge dates, discharge diagnoses and procedures (International Classification of Diseases, 9th Revision, Clinical Modification ICD-9-CM), wards of hospitalization, dates of in-hospital transfer, and the regional code of the admitting facility. To identify infant deliveries from the Hospital Information System, three different sources of information were used: the diagnosis-related group (DRG: 370–375), the procedure codes (ICD-9-CM: 72.x, 73.2, 73.5, 73.6, 73.8, 73.9, 74.0, 74.1, 74.2, 74.4, 74.99), and the diagnosis codes (ICD-9-CM: V27.xx, 640.xy - 676.xy, where y = 1 or 2). Cesarean deliveries were defined as DRG 370–371, procedure codes ICD-9-CM 74.0, 74.1, 74.2, 74.4, 74.99, and diagnosis code ICD-9-CM 669.7.

The proportion of CS deliveries was calculated as the ratio of caesarean deliveries to the total number of deliveries by women without a previous CS.

### Exclusion criteria

We excluded from the analysis all deliveries related to mothers that were not residents of Italy; mothers under the age of 10 or over the age of 55 years; hospital discharges with a stillbirth diagnosis (diagnosis codes ICD-9-CM: 656.4, V27.1, V27.4, V27.7). Moreover, all deliveries were excluded for mothers that had undergone a CS in the two years preceding the current delivery (diagnosis code ICD-9-CM 654.2; procedure codes ICD-9-CM: 74.0, 74.1, 74.2, 74.4, 74.99). Therefore, the analysis focused on *primary* CSs.

### Risk factors for cesarean section

Data on maternal and neonatal clinical factors that constitute indication for CS were collected based on primary and secondary discharge diagnoses from the Hospital Information System; information was retrieved from the hospitalization for delivery, and all hospital admissions in the previous two years. A detailed description of diagnoses and the associated ICD-9-CM codes is reported (see Additional file 1). Maternal ages were classified as: ≤ 17, 18–24, 25–28, 29–33, 34–38, and ≥ 39 years.

Malposition and malpresentation of the fetus were defined according to two distinct coding systems. The *standard definition* was ICD-9-CM 652; the *modified definition* was ICD-9-CM 652, but excluding 652.5 (HFH).

### Statistical analysis

The HFH prevalence and the difference in HFH prevalences between CS and vaginal delivery groups were evaluated for each hospital; we calculated the median, the 90^th^ and 95^th^ percentiles. The difference between HFH prevalences was calculated according to the following formula: the prevalence (%) of HFH in women that underwent a CS delivery *minus* the prevalence (%) of HFH in women that underwent a vaginal delivery. The statistical association between two variables was evaluated with the Spearman's rank correlation coefficient.

Geographical maps were produced to compare the prevalence of HFH and the difference in HFH prevalences between CS and vaginal delivery groups for each Local Health Unit, a body delegated by the National Health System to provide health care to a specific area. The classes used in the maps have been calculated applying the Jenks natural breaks optimization algorithm, which reduces the variance within classes and maximizes the variance between classes [17]. To evaluate the time-trend of HFH prevalences, percentages of HFH were calculated for each year (from 2005 to 2010), with respect to total deliveries, CS deliveries, and vaginal deliveries. The modified Poisson regression model for prospective studies with binary data was performed to estimate the adjusted, hospital-specific, proportions of CS deliveries [18]. Potential confounders were selected in two steps. In the first step, potential risk factors were selected on the basis of *a priori* knowledge of clinical characteristics that constitute an indication for CS; these included over 40 maternal and neonatal clinical factors. In the second step, the *a priori* risk factors were selected through a bootstrap stepwise procedure to determine which factors were actually associated with the outcome of interest (CS). These steps identified the predictive model [19]. With this approach, we selected 1000 replicated bootstrap samples from the original cohort. A bootstrap sample is a sample of the same size as the original dataset, where subjects are selected with replacement. Thus, a given subject in the original cohort may occur multiple times, only once, or not at all, in a specific bootstrap sample. A stepwise regression was performed in each replicated sample with thresholds of p = 0.05 for variable selection and for variable elimination [19]. Only risk factors selected in at least 50% of the procedures were finally considered to be potential confounders. Further details on the statistical methods were published elsewhere [20].

The *standard* and *modified* definitions of malposition and malpresentation of the fetus were considered in two separate bootstrap stepwise procedures. This gave rise to two different predictive models for the control of confounding factors.

After weighing advantages and disadvantages of random and fixed effects modeling, we preferred to use fixed-effects modeling to calculate hospital-specific prevalences. In fact, the random-effects analysis introduces a bias, the shrinkage towards the mean, because of which “high performing” hospitals are presented too negatively and “low performing” hospital too positively [21–23]. However, the lowest and highest proportions of both CS and HFH may have been recorded at low volume hospitals. In order to avoid the potential for extreme proportions, Empirical Bayes shrinkage estimator was applied as a sensitivity analysis [23].

The National Agency of Regional Health Services (Rome, Italy) gave approval for conducting this study.

## Results

A total of 540 maternity units were compared. Based on the Italian National Outcome Program results of 2010, the primary CS proportion was 28.3%.The highest primary CS proportions were found in the Campania region (47.5%), in the Sicily region (38.8%), and in the Calabria region (35.3%). The analysis of crude CS proportions showed a high variability among hospitals: values ranged from a minimum of 8.6% to a maximum of 91.6%. Using the Empirical Bayes Estimator, values ranged from 9.1% to 90.0%. The heterogeneity among maternity units was pronounced and significant: the variance component σ^2^_u0_ was equal to 0.5, with a standard error of 0.03 and a p-value less than 0.0001.

Table 1 shows the predictive model, including all the maternal and neonatal clinical factors selected by the bootstrap stepwise procedure as potential confounders. The area under the ROC curve was approximately 0.8. This model applied the standard definition of malposition and malpresentation of the fetus. This variable showed an adjusted Risk Ratio of 4.46 (95% CI: 4.42 - 4.50; p < 0.001).

**Table 1:** **Maternal and neonatal clinical factors (selected by the bootstrap stepwise procedure) included in the predictive model for CS (standard definition of malposition and malpresentation of the fetus), 2010**

		Prevalence ^a^ (%)	Crude risk ratio	Adjusted risk ratio	95% CI for Adj. RR	Adjusted P-value
Maternal age class	29–33 years (reference)	33.5	1.00	1.00	-	-
	≤ 17 years	0.6	1.16	1.10	1.05 - 1.16	< 0.001
	18-24 years	12.1	0.96	0.97	0.96 - 0.98	< 0.001
	25-28 years	17.8	1.00	0.99	0.98 - 1.01	0.361
	34-38 years	27.4	1.05	1.04	1.03 - 1.05	< 0.001
	39-55 years	8.5	1.34	1.22	1.20 - 1.24	< 0.001
Malignant tumors		< 0.1	2.47	2.30	1.84 - 2.88	< 0.001
Thyroid disease		0.4	1.45	1.16	1.08 - 1.24	< 0.001
Diabetes		0.1	2.21	1.70	1.49 - 1.93	< 0.001
Anemias		0.4	1.54	1.25	1.17 - 1.33	< 0.001
Coagulation defects		0.1	1.60	1.32	1.19 - 1.46	< 0.001
Hypertension		0.1	1.94	1.45	1.31 - 1.60	< 0.001
Heart disease		0.1	2.09	1.46	1.25 - 1.69	< 0.001
Acute pulmonary disease		< 0.1	2.31	1.72	1.39 - 2.13	< 0.001
Asthma		0.1	1.39	1.29	1.14 - 1.46	< 0.001
Cerebrovascular diseases		< 0.1	2.24	2.14	1.83 - 2.51	< 0.001
Collagen diseases		< 0.1	1.67	1.54	1.07 - 2.21	0.020
Congenital anomalies of the heart and circulatory system		0.1	1.72	1.28	1.05 - 1.57	0.015
HIV		0.1	3.24	3.69	3.29 - 4.15	< 0.001
Genital herpes		<0.1	3.02	3.75	2.47 - 5.70	< 0.001
Other Sexually Transmitted Diseases		< 0.1	1.61	1.41	1.10 - 1.81	0.007
Liver disorders in pregnancy		0.5	1.67	1.60	1.51 - 1.70	< 0.001
Cardiovascular disease in pregnancy		0.1	2.41	2.36	2.16 - 2.59	< 0.001
Renal disease in pregnancy		0.1	1.93	1.71	1.52 - 1.92	< 0.001
High-risk pregnancy		0.4	1.64	1.20	1.13 - 1.28	< 0.001
Antepartum hemorrhage, placenta previa		1.2	3.41	3.61	3.51 - 3.71	< 0.001
Eclampsia/pre-eclampsia		1.5	2.78	2.43	2.36 - 2.52	< 0.001
Multiple pregnancy		1.7	3.17	2.32	2.27 - 2.38	< 0.001
Malposition and malpresentation of the fetus		8.1	4.34	4.46	4.42 - 4.50	< 0.001
Excessive development of the infant		1.6	2.96	3.33	3.26 - 3.41	< 0.001
Fetal abnormality		0.6	2.03	2.06	1.96 - 2.16	< 0.001
Fetal distress		2.3	3.24	3.51	3.44 - 3.59	< 0.001
Intrauterine growth retardation		1.8	2.62	1.86	1.81 - 1.91	< 0.001
Pathology of the amniotic fluid		3.9	2.09	1.94	1.91 - 1.98	< 0.001
Premature rupture of membranes		10.2	0.81	0.93	0.91 - 0.94	< 0.001
Umbilical cord prolapse		0.1	3.21	2.45	2.13 - 2.81	< 0.001
Assisted fecundation		0.1	2.85	1.77	1.54 - 2.04	< 0.001

CS: caesarean section.

^a^The analysis was conducted on data from 423,090 women.

Area under the ROC curve = 0.799.

**Figure 1:**
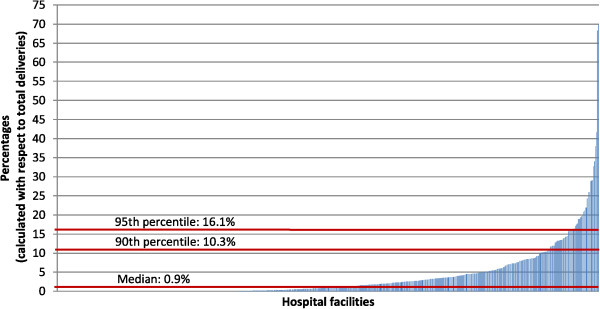
**Prevalence of HFH in 540 Italian hospitals, 2010.**

In Figure 1, all the considered maternity units were sorted, in ascending order, according to the prevalence of HFH. This fetal malposition was quite heterogeneously distributed among Italian health providers, with a median prevalence of 0.9% and a range of 0.0% to 69.8%. In 27 hospitals, the prevalence of HFH was greater than 16.1% (the 95^th^ percentile). Consistent results were obtained using the Empirical Bayes estimator: the median prevalence of HFH was 0.9%, the range was from 0.0% to 69.6%, the 95^th^ percentile was 16.0%. Moreover, the heterogeneity among maternity units was very high and statistically significant: the variance component σ^2^_u0_ was equal to 7.1, with a standard error of 0.6 and a p-value less than 0.0001.

**Figure 2:**
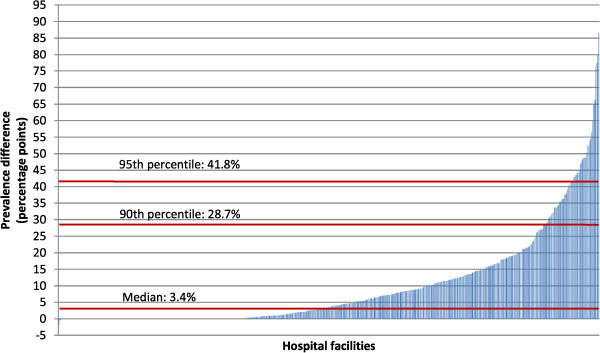
**Difference in HFH prevalences between caesarean section and vaginal delivery groups** ^**a**^ **for each of 540 Italian hospitals, 2010.** ^a^The difference in HFH prevalences was calculated with the following formula: prevalence (%) of HFH in women thatunderwent a cesarean section minus the prevalence (%) of HFH in women that underwent a vaginal delivery.

In Figure 2, the maternity units were sorted in ascending order according to the difference in HFH prevalences between CS and vaginal delivery groups. Again, great heterogeneity was observed among providers. The median difference in prevalence, expressed in percentage points, was 3.4, with a range of -2.1 to 86.6.

**Figure 3:**
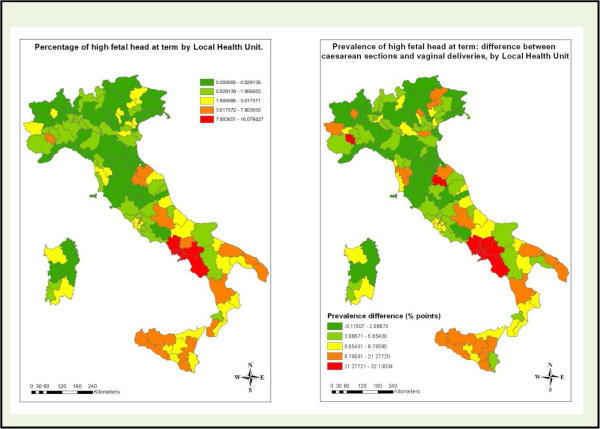
**Prevalence of HFH and the difference in HFH prevalences between caesarean sections and vaginal delivery groups for individual Italian Local Health Units, 2010.**

The geographical analyses presented in Figure 3 show the prevalence of HFH and the difference in HFH prevalences between CS and vaginal delivery groups for each Local Health Unit. Note that high values of both variables were concentrated in the Campania region. In this area, six out of seven Local Health Units had the highest proportions of HFH (>7.8%), and all seven Local Health Units had the highest difference in HFH prevalences (>21.3 percentage points).

**Figure 4:**
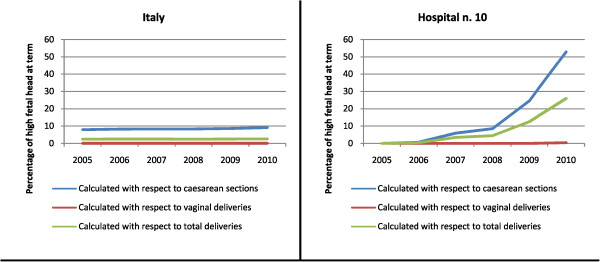
**Yearly percentages of HFH (2005–2010), calculated with respect to the total numbers of all deliveries, caesarean sections, and vaginal deliveries, in all of Italy and in Hospital number 10.**

The temporal analyses are shown in Figure 4. The time-course of HFH prevalence is shown for all of Italy and for a hospital in the Campania region that was among the 27 facilities with a HFH prevalence greater than the 95^th^ percentile. For each year, we calculated the percentages of HFH with respect to the numbers of all deliveries, CS deliveries, and vaginal deliveries. The overall national HFH prevalence remained constant over time (about 2.5%). In addition, over Italy, the difference in HFH prevalence between CS and vaginal delivery groups also remained constant over time. In contrast, the selected hospital showed an increasing trend in HFH prevalence with respect to the total number of deliveries from 2005 (0.0%) to 2010 (26.0%); this trend was totally driven by the increasing prevalence of HFH in women that underwent CS (0.0% to 52.9%).

With regard to the whole set of the 27 hospitals having HFH prevalences greater than the 95^th^ percentile, HFH prevalence has increased progressively from 2005 to 2010, showing the following trend: 14.6%, 16.2%, 16.6%, 17.4%, 21.9%, 25.6%.

**Figure 5:**
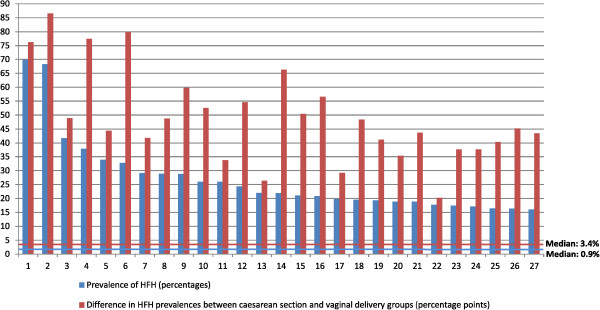
**Prevalence of HFH and the difference in HFH prevalences between caesarean sections and vaginal delivery groups for individual Italian hospitals** ^**a**^ **, 2010.** ^a^This Figure was restricted to focus on the 27 maternity units with HFH prevalence greater than the 95^th^ percentile.

Figure 5 shows the prevalence of HFH (in descending order) and the difference in HFH prevalences between CS and vaginal delivery groups for individual hospitals. Median values are also shown. This analysis was restricted to data from the 27 maternity units that showed HFH prevalences greater than the 95^th^ percentile. About 63% of these units were located in the Campania region. Of note, the maternity units with the highest HFH prevalences also had very large differences in HFH prevalences between CS and vaginal delivery groups. An analysis of the whole set of Italian hospitals (540 maternity units) showed a Spearman's rank correlation coefficient of about 97% for the association between these variables (the HFH prevalence and the difference in HFH prevalences between CS and vaginal delivery groups).

**Figure 6:**
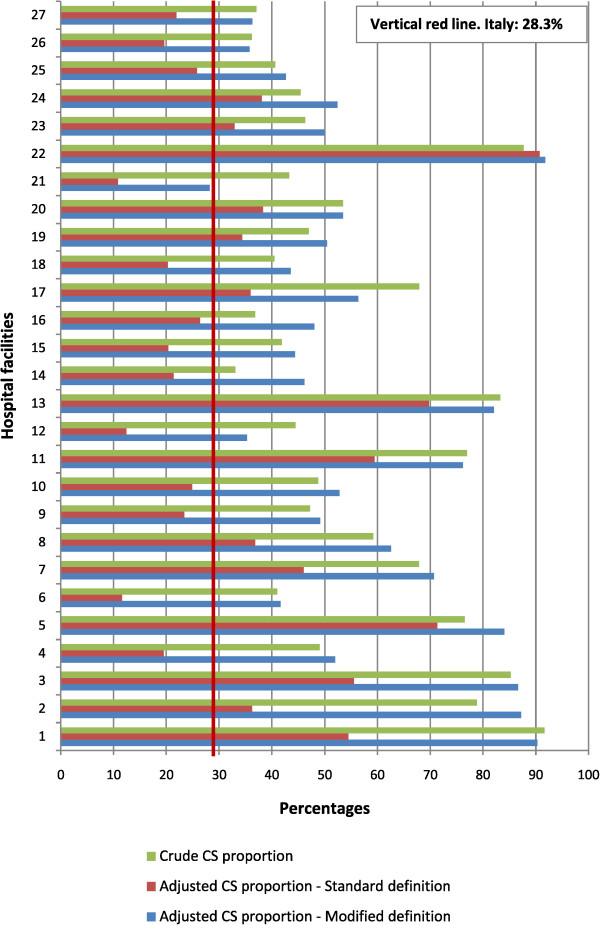
**Crude and adjusted primary CS proportions for 27 hospitals** ^**a**^ **; provide a comparison between two risk-adjustment models, one based on the standard definition and the other based on the modified definition of malposition and malpresentation of the fetus** ^**b**^ **(2010).** ^a^This Figure was restricted to focus on the 27 maternity units with HFH prevalence greater than the 95^th^ percentile. ^b^Standard definition of malposition and malpresentation of the fetus: ICD-9-CM 652. Modified definition: ICD-9-CM 652, excluding 652.5 (High fetal head at term).

The proportions of primary CS deliveries are shown for the 27 maternity units previously identified (Figure 6). All these hospitals had very high crude (unadjusted) proportions of CS deliveries. In some units, when the crude estimate was adjusted with the “*standard definition* model”, a relevant reduction was observed in the proportion of CS deliveries. In some cases, even starting with a very high crude proportion of CS, the adjustment resulted in a reduction so large that the hospital dropped below the national average (indicated by the vertical red line).

Very different results were observed when the risk-adjustment procedure was performed with the *modified definition* of malposition and malpresentation of the fetus (which excluded HFH). Replacing the *standard definition* with the *modified definition* in developing the predictive model it’s adjusted risk ratio decreased from 4.46 to 4.02 (95% CI: 3.98 - 4.07; p < 0.001). For nearly all the 27 hospitals, the adjusted estimates of the proportion of CS obtained with the “*modified definition* model” were substantially higher than those obtained with the “*standard definition* model”. Therefore, in contrast to the pronounced reduction observed after adjusting the crude estimates with the “*standard definition* model”, we observed little or no reduction after adjusting with the “*modified definition* model”. Conversely, we selected a random sample of maternity units that behaved in a “virtuous manner” (defined as having percentages of HFH in women that underwent CS delivery less than or equal to the analogous national percentage). When comparing the “*standard definition*” to the “*modified definition*”, no substantial differences were observed between the resulting risk-adjusted CS proportions.

## Discussion

This study compared hospitals on the basis of the proportions of CS deliveries, with the implicit assumption that lower rates reflect a more appropriate practice. We found that differential confounder misclassification may lead to unpredictable consequences and misleading results.

Our investigation focused on malpositions and malpresentation of the fetus, one of the most important indications for performing a CS. In the predictive model, this factor had the highest risk ratio, with p < 0.001. Among the different fetal malpositions and malpresentations, we focused on one specific condition, HFH. In fact, because this condition is seldom objectively documentable in medical records, its reliability is difficult to verify in case of clinical audit; therefore, HFH may be subject to improper and opportunistic coding. The prevalence of HFH (median 0.9%) was heterogeneously distributed among Italian health providers, with a range of 0.0% to 69.8%. Very close results were obtained using the Empirical Bayes estimator probably because, in the our cohort, the hospital sample size was generally high: estimates in large group are more reliable, and shrink less than estimates from small groups. In some maternity units, HFH was coded in such a large number of cases that it was unlikely to reflect the natural variability of the phenomenon. This raised the reasonable hypothesis that HFH may be particularly subject to misclassification, which was differential with respect to the exposure status (in this case, represented by the maternity unit). Similarly, the difference in HFH prevalences between CS and vaginal delivery groups also showed great heterogeneity among health care providers. The difference in HFH prevalence (median 3.4 percentage points) ranged from -2.1 to 86.6. Thus, HFH misclassification may also have been differential with respect to the outcome (the type of delivery, cesarean or vaginal). In fact, in some maternity units, HFH over-coding occurred more frequently for women that had undergone CS. This suggested that over-coding was due to opportunistic behavior in reporting factors that are an indication for CS delivery, in an attempt to justify the choice of a surgical procedure. These findings were promptly reported to the Italian Ministry of Health, which has implemented a system of validation sub-studies to evaluate the actual rates of misclassification of HFH. However, the results have not yet been disclosed by the Ministry. Validation studies have been carried out throughout the country analyzing a large random sample of maternity units, oversampling in particular areas.

In fact, the geographical analysis showed that HFH misclassification was not randomly distributed over Italy, but it was markedly “excessive” in the Campania region. In 2005, the local government of Campania, in an attempt to reduce the high number of CS deliveries, enacted a series of regional regulations to promote the appropriateness of the CS; in 2007, the Campanian government deliberated that remuneration would be paid only for CSs justified by the presence of maternal or neonatal risk factors. Many maternity units in the Campania region have “adapted” to this regulation; thus, the proportion of HFH codes gradually increased from 2005 to 2010, though the national average remained constant.

In general terms, in some maternity units, the high proportion of CS appeared to “cause” a high proportion of HFH; in other words, the high proportion of HFH was totally driven by the *a posteriori* coding of HFH in women that had undergone CS deliveries. This hypothesis could explain the high correlation between the prevalence of HFH and the difference in HFH prevalences observed between CS and vaginal delivery groups.

We focused on the 27 maternity units that had HFH prevalences greater than the 95^th^ percentile. This selection facilitated an evaluation of the impact that a differential misclassification might have on the hospital-specific risk-adjusted proportions. When a diagnosis is falsely codified to justify a procedure, it cannot continue to be considered a potential confounder, because it is an artifactual effect of the outcome, which was the CS, in this case [1]. The inclusion of such factors in risk-adjustment models compromises the validity of the estimates and favors those hospitals that behaved in a less-than-virtuous manner. Therefore, our findings suggested that close attention should be given to health care quality risk-adjusted comparisons over time and space. In fact, the validity of confounder classifications may be subject to substantial variation both over time (as occurred in the Campania region) and over different geographical areas.

In the specific case of the CS, the bias appeared to be reduced with the use of the *modified definition* of malposition and malpresentation of the fetus, which omitted HFH. In fact, in the majority of cases, the pronounced reduction of the crude CS proportion after adjustment was eliminated or ameliorated by removing the HFH observation. However, this solution may not be definitive, because some hospitals may use other types of opportunistic coding, which are difficult to predict *a priori*. Thus, all confounding factors should be carefully inspected before proceeding with any comparative evaluation. In this perspective, it is worth noting the opportunity of applying other methods to mitigate this particular kind of bias. One of the most interesting solutions is the Quantitative bias analysis, which provides a methodology for assessing the impact of bias on study results by making assumptions about the bias parameters, in order to analytically address the problem of differential misclassification [24].

## Conclusion

Differential misclassification of confounders in comparative evaluations of hospital care may lead to unpredictable consequences and misleading results. We focused on the appropriateness of CS, and we found that some malpositions and malpresentations of the fetus (such as HFH) may be deliberately misclassified to justify a CS procedure in the absence of actual risk factors. This practice generated a differential misclassification and favored those hospitals that behaved in a less-than-virtuous manner. Our findings suggested that close attention should be given to inspecting for differential misclassification of confounders, because their validity may be subject to substantial variation over hospitals, over time and over different geographical areas. This kind of bias may largely mislead public and local health policies aimed at improving the quality of hospital care.

### Consent

In this retrospective, observational, public health study no interventions were performed. Results were provided in aggregate form only. Investigators has made adequate provisions to protect privacy, assure confidentiality of data, and respect the subject's rights, according to the Helsinki Declaration's ethical standards.

## Electronic supplementary material

Additional file 1: **Risk factors for caesarean section.** (DOCX 14 KB)

## Abbreviations

CS: Caesarean sections
HFH: High fetal head at term.
