# Supplementary material for: Differential misclassification of confounders in comparative evaluation of hospital care quality: caesarean sections in Italy
Source: BMC Public Health. 2014 Oct 8;14:1049. doi: 10.1186/1471-2458-14-1049 (PMC4210510; doi:10.1186/1471-2458-14-1049)
Supplement: Supplementary file 1 — Additional file 1: Risk factors for caesarean section. (DOCX 14 KB) [file 12889_2013_7175_MOESM1_ESM.docx]

**Additional file 1**. Risk factors for caesarean section

|  | **ICD-9-CM codes** | |
| --- | --- | --- |
| **Risk factors** | **Hospitalization for delivery** | **Hospital admissions in the previous two years** |
| Malignant tumors | 140.0–208.9, V10 | 140.0–208.9, V10 |
| Thyroid disease | 240-246, 648.1 | 240-246 |
| Diabetes | 250.0-250.9, 648.0 | 250.0-250.9 |
| Anemias | 280-284, 285 (excluding 285.1), 648.2 | 280-284, 285 (excluding 285.1) |
| Coagulation defects | 286 | 286 |
| Hypertension | 401-405, 642.0-642.3, 642.9 | 401-405 |
| Heart disease | 390-398, 410-429 | 390-398, 410-429 |
| Acute pulmonary disease | 480-487, 510-514 | - |
| Chronic pulmonary disease | 500-508, 515-517 | 500- 508, 515-517 |
| Asthma | 493 | 493 |
| Cerebrovascular diseases | 433, 437, 438 | 430-432, 433, 434, 436, 437, 438 |
| Collagen diseases | 710 | 710 |
| Congenital anomalies of the heart and circulatory system | 745-747 | 745-747 |
| Nephritis, nephrotic syndrome, and nephrosis | 580-589 | 580-589 |
| Chronic obstructive pulmonary disease | 491-492, 494, 496 | 491-492, 494, 496 |
| Cystic fibrosis | 277.0 | 277.0 |
| Tuberculosis | 010-018, 647.3 | 010-018 |
| HIV | 042, 079.53, V08 | 042, 079.53, V08 |
| Genital herpes | 054.1 | - |
| Other Sexually Transmitted Diseases | 077.98, 078.88, 079.88, 079.98, 090-099, 647.0- 647.2 | - |
| Liver disorders in pregnancy | 646.7 | - |
| Cardiovascular disease in pregnancy | 648.5, 648.6 | - |
| Renal disease in pregnancy | 646.2 | - |
| Substance abuse | 303-305; 648.3 (excluding 648.32 e 648.34) | - |
| Maternal diseases affecting fetus or newborn | 760.0, 760.1, 760.3 | - |
| High-risk pregnancy | 640, 644.0, V23.0, V23.2, V23.4, V23.5, V23.7, V23.8 | - |
| Antepartum hemorrhage, abruptio placentae, placenta previa | 641 | - |
| Eclampsia/pre-eclampsia | 642.4-642.7 | - |
| Multiple pregnancy | 651, V27.2–V27.9, V31-V37, 761.5 | - |
| Malposition and malpresentation of the fetus (*standard definition*) | 652 | - |
| Fetopelvic disproportion/excessive development of the infant | 653, 656.60, 656.61, 656.63 | - |
| Fetal abnormality | 655 | - |
| Fetal distress | 656.3, 768 | - |
| Intrauterine growth retardation | 656.5, 764 | - |
| Pathology of the amniotic fluid | 657, 658.0, 658.4 | - |
| Premature rupture of membranes | 658.1 | - |
| Umbilical cord prolapse | 663.0 | - |
| Pre-term Birth | 644.1, 644.2 | - |
| Post-term birth | 645 | - |
| RH-Isoimmunization | 656.1 | - |
| Assisted fecundation | V26 | - |
